# Anti-cancer activity of new benzyl isoquinoline alkaloid from Saudi plant *Annona squamosa*

**DOI:** 10.1186/s13065-019-0536-4

**Published:** 2019-02-04

**Authors:** Adel M. Al-ghazzawi

**Affiliations:** Department of Chemistry, King Khalid University, Abha, 61413 Kingdom of Saudi Arabia

**Keywords:** *Annona squamosa*, Annonacea, (6, 7-dimethoxy-1-(α-hydroxy-4-methoxybenzyl)-2-methyl-1, 2, 3, 4-tetrahydroisoquinolin, Coclaurine

## Abstract

Two alkaloids, belonging to benzylisoquinoline alkaloids, were isolated from *Annona squamosa*. One of these alkaloids, 6, 7-dimethoxy-1-(α-hydroxy-4-methoxybenzyl)-2-methyl-1, 2, 3, 4-tetrahydroisoquinoline, was isolated for the first time from natural sources, while, the alkaloid, Coclaurine was known in *Annona squamosa* L. Moreover, the isolated alkaloids tested for the anti-cancer activities on various cell lines (HepG-2, MCF-7, and HCT-116).

**Electronic supplementary material:**

The online version of this article (10.1186/s13065-019-0536-4) contains supplementary material, which is available to authorized users.

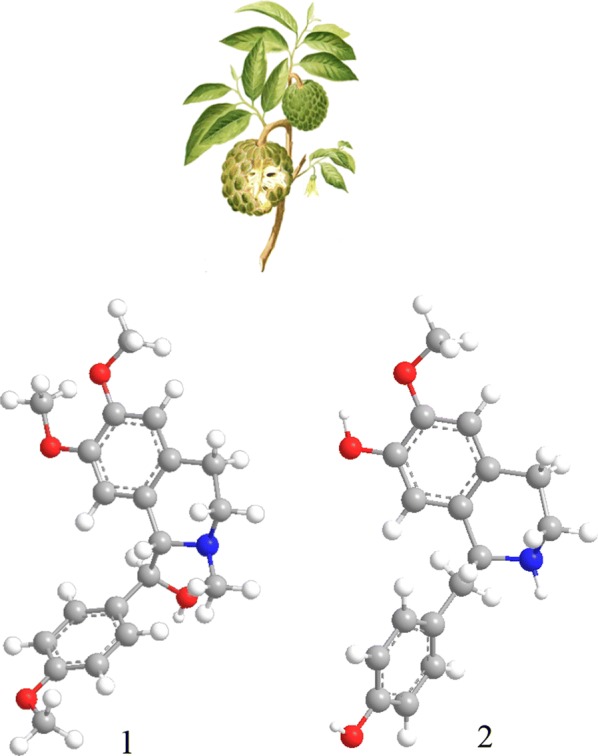

## Introduction

*Annona* L. belongs to the family Annonaceae which is a large family comprising about 135 genera and more than 2500 species distributed mainly in tropical and sub-tropical regions [1–3]. *Annona* genus includes approximately 162 species of trees, shrubs and, rarely lianas [4]. Some species of *Annona* are of economic importance because of their edible fruits and medicinal properties, like *Annona squamosa* (sugar apple), *Annona muricata* (soursop), *Annona reticulata* (custard-apple) and *Annona cherimola* (cherimoya) [2]. Only one of *Annona* genus *Annona squamosa* was reported in Saudi Arabia [4]. *Annona squamosa* small tree, 2–3 m tall. Leaves without stipules, petiolate, alternate, lanceolate to elliptic-oblong, 8–13 × 3–6 cm, entire. Flowers solitary or in clusters of 2–4, arising opposite the leaves, borne on a recurved pedicel. Perianth segments in 3 s. Narrowly triangular, green to yellowish brown. Stamens were numerous. Carpels united into a fleshy mass in fruit. Seeds brown, surrounded by white, sweet pulp [4, 5].

Traditionally, all parts of *A. squamosa* are used by different ethnic communities for the treatment of various chronic diseases such as cancerous tumors, insect bites and other skin complaints [5–8]. However, the seeds powder is toxic and used to kill head lice and fleas [5, 9]. The leaves used for a long time as ant- diabetics, anti-ulcer, anti-depressants, anti-inflammatory, antimicrobial and antifungal [10–15]. It has also used as Immunomodulatory and hepatoprotective [5, 9, 16]. Also, it used as fertility control [17].

Constituents of *Annona squamosa* have chemical compounds approximately belongs to all natural products compounds steroid, terpenoids, glycoside, alkaloid, flavonoid saponin and phenolic compounds [5, 9, 16].

All previous study done on the anti-cancer activity of *A. squamosa* were dealing with the crude extract that contain all chemical constituents of *A. squamosa* or with non-alkaloidal parts especially acetogenin [18, 19]. Because of there are several natural products have which have anti-cancer activity contain N-atom in there skeleton reported in literature [20–22] and no previous study dealing with the anticancer activity of alkaloids part of *A. squamosa*, so in this research, we isolate some pure alkaloids from this plant also, we study the anti-cancer activity of the isolated alkaloids.

## Results and discussion

### Chemical analysis of Annona squamosal

**Fig. 1:**
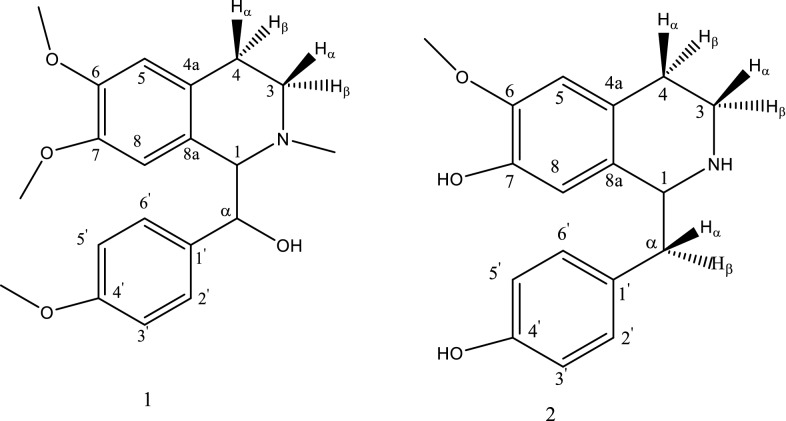
Chemical structure of 1 and 2

Chemical investigation of alkaloidal part of *A. squamosa* from Saudi origin resulted in the isolation of two benzylisoquinoline alkaloids, namely6, 7-dimethoxy-1-(α-hydroxy-4-methoxybenzyl)-2-methyl-1, 2, 3, 4-tetrahydroisoquinolin.1 and Coclaurine 2 (Fig. 1). The first one isolated for the first time from nature, while coclaurine were isolated before from *A. squamosa*.

### Structure elucidated for the isolated compounds

#### 6, 7-dimethoxy-1-(α-hydroxy-4-meth oxybenzyl)-2-methyl-1, 2, 3, 4-tetrahydroisoquinolin (I)

The ^1^H-NMR of compound (I) (Table 1, see Additional file 1) shows two doublets in the aromatic region each one integrating for two protons which indicates para-substituted benzene ring. The first doublet resonates at δ = 7.028 ppm (J = 10.5 Hz) assigned for 2′ and 6′ while; another doublet appears at δ = 6.838 ppm (J = 10.5 Hz) assigned for 3′ and 5′. Also, two singlet appears at δ = 6.838 ppm and δ = 6.649 ppm assigned for H-5 and H-8 respectively. The down-filled shift of H-8 due to the inter hydrogen bonding between the hydroxyl group and the tertiary nitrogen which was confirmed by the IR spectroscopy. The hydroxyl methine proton resonates at δ = 5.610 ppm as broad singlet while the proton of hydroxyl group appears as a broad singlet at δ = 5.171 ppm. The protons for C-3 and C-4 overlapped between 3-4 ppm. The spectrum shows two singlets integrating for 3 protons at δ = 3.884 ppm and δ = 3.798 ppm for methoxy group at C-6 and C-4′ respectively moreover, the intermolecular hydrogen bonding between the hydroxyl group and the tertiary nitrogen can affect the protons of methoxy group at C-7 which is downfield shifted to δ = 3.468 ppm. The N-methyl protons resonate as a singlet at δ = 2.885 ppm. The ^13^C-NMR (see Additional file 1) shows 13 peaks, 7 of them for aromatic carbons, 3 peaks resonate at δ = 55.455, 55.326 and 55.906 ppm assigned for methoxy carbon, the *N*-methyl carbon appears at δ = 40.046 ppm also, the DEPT experiment confirms the presence of 5 methyl groups, four methines in aromatic region and 1 methylene carbon, too, one methine appears in upfield region resonate at δ = 77.230 ppm.

**Table 1:** ^1^H and ^13^C NMR spectroscopic data for 1and 2

No.	1^a^		2^b^	
	δ_C_ type	δ_H_ mult. (J)	δ_C_ type	δ_H_ mult. (J)
1	77.230	5.610 br s	55.157	4.427 m
3	d	3.00–4.00 m	38.95	3.35, 3.25 c
4	29.715	3.00-4.00 m	24.88	2.962, 2.858 m
4a	d	–	122.72	–
5	111.109	6.849 s	115.35	6.727 s
6	147.196	–	147.05	–
7	149.079	–	144.81	–
8	110.966	6.649 s	11.85	6.563 s
8a	d	–	124.72	–
α	d	–	38.79	3.152,3.025dd (18,8)
1^′^		–	126.31	–
2^′^	131.342	7.028 d (10.5)	130.566	7.132 d (10)
3^′^	114.072	6.838 d (10.5)	111.813	6.786 d (10)
4^′^	158.906	–	156.33	–
5^′^	114.072	6.838 d (10.5)	111.813	6.786 d (10)
6^′^	131.342	7.028 d (10.5)	130.566	7.132 d (10)
6-OCH_3_	55.455	3.884 s	55.526	3.752 s
7-OCH_3_	55.326	3.798 s	–	–
4^′^-OCH_3_	55.906	3.468 s	–	–
N-CH_3_	40.046	2.885 s	–	–

^a^ Data were recorded in CDCl_3_ 500 MHz (1H) and 75 MHz (^13^C)

^b^ Data were recorded in DMSO-d_6_ at 500 MHz (^1^H) and 75 MHz (^13^C)

^c^ Overlapped

^d^ Not detected but appears in HMBC

**Fig. 2:**
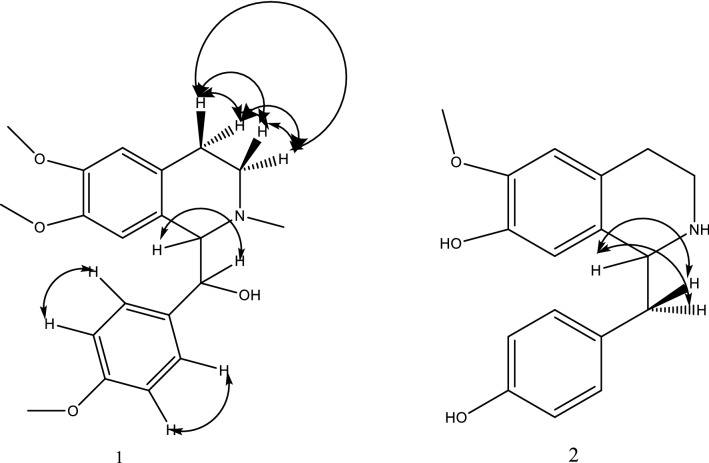
COSY correlations of compounds 1 and 2

The COSY experiment shows a good correlation between the C-3 protons and the C-4 protons, also, good correlations between para-substituted benzene ring protons. The most significant correlation is between C-1 proton and C-α proton (Fig. 2).

**Fig. 3:**
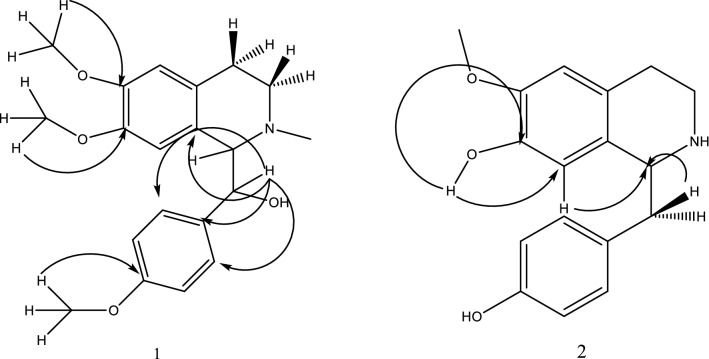
HMBC correlations of compounds 1 and 2

The chemical shifts of the different carbons of compound 1 assigned with the help of HMQC and HMBC experiments. The HMBC shows some good correlations between the methoxy protons and the aromatic carbons to which the methoxy groups are attached. Also, shows a good correlation between the protons of C-α and C-1′, C-2′, C-6′and C-8 (Fig. 3).

#### Coclaurine

The ^1^H-NMR of compound (I) (Table 1, see Additional file 1) shows two singlets resonate at δ = 6.727 ppm and δ = 6.563 ppm assigned for C-5 and C-8 respectively. Also, two doublets each integrating for 2 protons appears at δ = 7.132 ppm (J = 10 Hz) and δ = 6.786 ppm (J = 10 Hz) indicating a para disubstituted benzene ring assigned for protons of ring C. The C-1protons resonate at δ = 4.427 ppm as multiplet while, the two protons of C-α appears as two doublets at δ = 3.152 ppm (J = 18, 8 Hz) and at δ = 3.025 ppm (J = 18,10 Hz). The first coupling due to geminal coupling between the C-α while the second coupling with the C-1 proton.

The ^13^C-NMR spectra (see Additional file 1) show 15 peaks for 17 C-atom indicting the presence of para disubstituted benzene ring in the compounds the DEPT experiments revealed this since the DEPT 135 and DEPT 90 shows four tertiary carbons and six quaternary carbons in aromatic region. Also, shows one methyl, two methylene and one methine carbons in aliphatic region.

The COSY experiments show good correlations between the two C-α protons and C-1 proton (Fig. 2).

The chemical shifts of the different carbons of compound 2 assigned with the help of HMQC and HMBC experiments. The HMBC shows good correlations between O–H proton which resonate at δ = 9.447 ppm with C-4′ and both of symmetric carbons C-3′ and C-5′. On the other hand, another O–H proton shows a good correlation with C-7 and C-8 which indicate that the hydroxyl group is attached to C-7 rather than C-6. Also, one of the important correlations seen in HMBC spectra is the correlation between the C-1 carbon and the proton of C-8 and protons of C-α (Fig. 3).

### Anti-cancer activity

Anti-cancer activity of *Annona* plants reported in many documents, here, in this research, we study the anti-cancer activity of purely isolated alkaloids. In our study, we use three types of cancer cell line namely: Human Colon cancer cells (HCT116), Human Brest cancer cells (MCF-7) and Human Liver cancer cells (HEPG-2). Table 2 shows the IC50 of Coclaurine and compound 2 against the mentioned cell line. The two isolated compounds gave an excellent activity on the three cell line; also, the two compounds show the most activity against HepG-2, but coclaurine shows a better activity than compound 2 this result on isolated compounds is in confident with structure–activity relationships studies of anti-cancer activity of benzylisoquinoline alkaloids. The SAR studies show that the increase in the number of hydroxyl groups in the BIQ alkaloids increase the anti-cancer activity, on the other hand, methylation of nitrogen atom decrease the anti-cancer activity [22].

**Table 2:** IC50 of tested compounds

IC50 µg/mL	Tested extract (compound)		
	Colon cancer cells (HCT116)	Human brest cancer cells (MCF-7)	Human liver cancer cells (HEPG-2)
Coclaurine	8.233	15.345	1.674
Compound 2	12.344	21.586	5.195
Doxorubicin	0.8105	1.358	0.777

## Conclusion

Chemical analysis of the alkaloidal part of *A. squamosa*, afforded two alkaloids belong to simple benzylisoquinoline alkaloid class. One of them is reported from natural sources for the first time.

Isolated alkaloids gave an excellent activity on Colon cancer cells (HCT116) and Human Brest cancer cells (MCF-7), which is confident with the reported structure–activity relationship of activity of benzylisoquinoline alkaloids on a cancer cell.

This result supports using the plant in folk medicine to treat cancer.

Authors recommended to make total synthesis of the isolated alkaloids.

## Materials and methods

### Chemicals and materials

All chemicals were purchased from Sigma-Aldrich. cell culture vessels were supplemented from Nunc Co. (Roskilde, Denmark). Human colon (HCT 116), Human liver (HepG-2) and Human breast (MCF-7) cancer cell lines were purchased from Vacsera (Giza, Egypt). Cells were maintained routinely in RPMI 1640 cell culture media supplemented with 1 mM sodium pyruvate, 2 mM L. glutamine, 100 units/mL penicillin–streptomycin and 10% fetal bovine serum. Cells were incubated in a humidified, 5% CO_2_ atmosphere at 37 °C.

^1^H-NMR spectra were recorded on a Bruker DPX-500 MHz spectrometer with TMS as an internal standard. ^13^C–NMR spectra were recorded at 125.8 MHz using the same instrument.

### Plant material

The aerial parts *A. squamosa*, Annonaceae, were collected from Jizan region in Kingdome of Saudi Arabia in January 2017. The plant material was identified in biology department of King Khalid University.

Preparation of plant material:

The aerial parts of the plant were dried in the shade for 15 days then ground to get 6 kg fine powder. The powder was soaked in petroleum ether of 10 days for defatting then extracted thoroughly with ethanol four times each time needs 7 days later the ethanol was evaporated to get a 650 g residue.

### Preparation of alkaloidal extract

The ethanol residue was dissolved in 5% HCl until the PH = 2 of the solution and filtered, the precipitate which contains neutral material was kept for further fractionation, and the filtrate which provides the basic material was basify using NH4OH solution, and the PH of the solution becomes around 8. After that, the solution was extracted with chloroform 500 mL three times the chloroform layer was evaporated to get 7.35 g of crude alkaloids which represent 0.123% of the dry plant.

### Chromatography of crude extract

The crude alkaloids were subjected to silica gel column chromatography using a column packed in chloroform and polarity increasing using methanol till pure methanol was used. The fractions collected (60 fractions, 0.25 L each) were grouped according to their TLC behavior into six groups. Fraction III gave upon treatment of methanol a yellowish amorphous solid I (30 mg). Fraction V gave a dark brown amorphous solid when treated with methanol this solid were recrystallize by methanol to provide a solid white II (50 mg).

### Physical and spectral data of isolated compounds from *Annona sequamosa*

*Compound I*: Yellowish amorphous solid, IR (KBr) ν_max_ (cm^−1^) : 3393, 2927, 2852, 1612, 1514. ^1^H-NMR and ^13^C-NMR data in Table 1.

*Coclaurine*: Compound II was crystallised from MeOH as white powder, m.p. 254–256d  °C, ^1^H-NMR and ^13^C-NMR data in Table 1.

### Drug dose preparation

0.01 g of each pure compounds was diluted in 1 mL of (DMSO) dimethyl sulfoxide as a stock solution.

### Anti-cancer activity of isolated compounds from *Annona sequamosa*

In the present study, SulphoRhodamine-B (SRB) assay had been chosen to detect the anticancer activity of isolated alkaloids. The anticancer activity of isolated alkaloids was tested against Human breast (MCF-7), Human colon (HCT 116), and Human liver (HepG-2) cancer cell lines. Cancer cells were exposed to a range of concentrations (0.01 to 100 µg/mL) of alkaloids and incubated in 5% CO_2_ humidified incubator at 37 °C for 72 h. Doxorubicin was used as a positive control. Cells were treated with the extracts for 72 h then; they were fixed with TCA (10%) for 1 h at 4 °C. To remove TCA cells were washed many times, then 0.4% SRB solution was used to stain cells in a dark place for 10 min. Stained cells were washed with 1% glacial acetic acid. Finally, to dissolve SRB-stained cells, Tris–HCl was used. After drying overnight, the color intensity of remained cells was measured at 540 nm by Elisa.

### Statistical analysis

The IC_50_ calculation was performed using Sigma Plot version 12.0

## Additional file

**Additional file 1: Figure S1.** ^13^C-NMR spectra of 6, 7-dimethoxy-1-(α-hydroxy-4-methoxybenzyl)-2-methyl-1, 2, 3, 4-tetrahydroisoquinoline.** Figure S2.** ^13^C-DEPT90 spectra of 6, 7-dimethoxy-1-(α-hydroxy-4-methoxybenzyl)-2-methyl-1, 2, 3, 4-tetrahydroisoquinoline.** Figure S3.** ^13^C-DEPT135spectra of 6, 7-dimethoxy-1-(α-hydroxy-4-methoxybenzyl)-2-methyl-1, 2, 3, 4-tetrahydroisoquinoline.** Figure S4. **^1^H-NMR spectra of 6, 7-dimethoxy-1-(α-hydroxy-4-methoxybenzyl)-2-methyl-1, 2, 3, 4-tetrahydroisoquinoline.** Figure S5.** ^13^C-NMR spectra of Coclaurine.** Figure S6.** ^13^C-DEPT 90 spectra of Coclaurine.** Figure S7.** ^13^C-DEPT 135 spectra of Coclaurine.** Figure S8.** ^1^H-NMR spectra of Coclaurine

## References

[1] Leboeuf M, Cave A, Bhaumik PK, Mukherjee B, Mukherjee R (1982). The phytochemistry of the annonaceae. Phytochemistry.

[2] Rabêlo SV, Costa EV, Barison A, Dutra LM, Nunes XP, Tomaz JC, Oliveira GG, Lopes NP, Fátima Md, Santos C, da Silva Almeida JRG (2015). Alkaloids isolated from the leaves of atemoya (*Annona cherimola* ×* Annona squamosa*). Revista Brasileira de Farmacognosia..

[3] Teles MNO, Dutra LM, Barison A, Costa EV (2015). Alkaloids from leaves of *Annona salzmannii* and Annona vepretorum (Annonaceae). Biochem Syst Ecol.

[4] Alfarhan AAH, Al-Turki TA, Basahy AY (2005) Flora of Jizan region. Final report, vol 1

[5] Saha R (2011). Pharmacognosy and pharmacology of *Annona squamosa*: a review. Int J Pharm Life Sci.

[6] Kadali VN, Pola SR, Sandeep BV (2016). Anti-cancer properties of plants in west Godavari district of Andhra Pradesh, India. Int J Pharmacogn.

[7] Vantitha V, Umadevi KJ, Vijayalakshmi K (2016). In vitro anti-proliferative effect of *Annona squamosa* (L.) leaf in the regulation of apoptotic genes in HEPG2 cell line. Int J Pharma Bio Sci.

[8] Chen Y, Chen Y, Shi Y, Ma C, Wang X, Li Y, Miao Y, Chen J, Li X (2016). Antitumor activityof *Annona squamosa* seed oil. J Ethno Pharmacol.

[9] Pandey N, Barve D (2011). Phytochemical and pharmacological review on *Annona squamosa* Linn. Int J Res Pharm Biomed Sci.

[10] Shirwaikar A, Rajendran K, Kumar CD, Bodla R (2004). Antidiabetic activity of diabetic rats. J Ethnopharmacol.

[11] Yadav CK, Singh N, Dev K, Sharma R, Sahai M, Palit G, Maurya R (2011). Anti-ulcer constituents of *Annona squamosa* twigs. Fitoterapia.

[12] Chavan MJ, Wakte PS, Shinde DB (2010). Analgesicandanti-inflammatory activity of Caryophyllene oxide from *Annona squamosa* L. bark. Phytomedicine.

[13] Jagtap UB, Bapat VA (2012). Antioxidant activities of various solvent extracts of custard apple (*Annona squamosa* L.) fruit pulp. Nutrafoods.

[14] Gupta RK, Kesari AN, Murthy PS, Chandra R, Tandon V, Watal G (2005). Hypoglycemic and antidiabetic effect of ethanolic extract of leaves of *Annona squamosa* L. in experimental animals. J Ethnopharmacol.

[15] Soni VK, Pathak M, Yadav DK, Maurya R, Sahai M, Jain SK, Bhattacharya SM (2013). Immunomodulatory constituents from *Annona squamosa* twigs provoke differential immune response in BALB/c mice. Curr Sci.

[16] Damasceno DC, Volpato GT, Sartori TCF, Rodrigues PF, Perin EA, Calderon IMP, Rudge MVC (2002). Effects of *Annona squamosa* extract on early pregnancy in rats. Phytomedicine.

[17] Zhou D, Sun LR, Feng F, Mo J, Zhu H, Yang B, He Q, Gan L (2013). Cytotoxic Diterpenoids from the Stem Bark of *Annona squamosa* L.. Helv Chim Acta.

[18] Miao Y, Shi Y, Li F, Shan C, Chen Y, Chen J, Li X (2016). Metabolomics study on the toxicity of *Annona squamosa* by ultra-performance liquid chromatography high-definition mass spectrometry coupled with pattern recognition approach and metabolic pathways analysis. J Ethnopharmacol.

[19] Reddy CR, Reddy MD, Dilipkumar U (2014). Total synthesis of a pyrrole lactone alkaloid, longanlactone. Eur J Org Chem.

[20] Reddy CR, Dilipkumar U, Reddy MD, Rao NN (2013). Total synthesis and revision of the absolute configuration of seimatopolide B. Org Biomol Chem.

[21] Venkateshwarlu R, Chinnababu B, Ramulu U, Reddy KP, Reddy MD, Sowjanya P, Rao PV, Aravind S (2017). Synthesis and biological evaluation of (−)- kunstleramide and its derivatives. Med Chem Commun.

[22] Cui W, Iwasa K, Tokuda H, Kashihara A, Mitani Y, Hasegawa T, Nishiyama Y, Moriyasu M, Nishino H, Hanaoka M, Mukai C, Takeda K (2006). Potential cancer chemopreventive activity of simple isoquinolines, 1-benzylisoquinolines, and protoberberines. Phytochemistry.

