# Supplementary material for: Anti-cancer activity of new benzyl isoquinoline alkaloid from Saudi plant Annona squamosa
Source: BMC Chem. 2019 Feb 4;13(1):13. doi: 10.1186/s13065-019-0536-4 (PMC6661725; doi:10.1186/s13065-019-0536-4)
Supplement: Supplementary file 1 — Additional file 1: Figure S1. 13C-NMR spectra of 6, 7-dimethoxy-1-(α-hydroxy-4-methoxybenzyl)-2-methyl-1, 2, 3, 4-tetrahydroisoquinoline. Figure S2. 13C-DEPT90 spectra of 6, 7-dimethoxy-1-(α-hydroxy-4-methoxybenzyl)-2-methyl-1, 2, 3, 4-tetrahydroisoquinoline. Figure S3. 13C-DEPT135spectra of 6, 7-dimethoxy-1-(α-hydroxy-4-methoxybenzyl)-2-methyl-1, 2, 3, 4-tetrahydroisoquinoline. Figure S4. 1H-NMR spectra of 6, 7-dimethoxy-1-(α-hydroxy-4-methoxybenzyl)-2-methyl-1, 2, 3, 4-tetrahydroisoquinoline. Figure S5. 13C-NMR spectra of Coclaurine. Figure S6. 13C-DEPT 90 spectra of Coclaurine. Figure S7. 13C-DEPT 135 spectra of Coclaurine. Figure S8. 1H-NMR spectra of Coclaurine [file 13065_2019_536_MOESM1_ESM.docx]

**Anti-cancer activity of new benzyl isoquinoline alkaloid from Saudi plant *Annona squamosa***

**Adel M. Al-ghazzawi^1^**

**Department of Chemistry, King Khalid University, Abha, 61413, Kingdome of Saudi Arabia**

^*^Corresponding author, e-mail address: [algawazy@kku.edu.sa](mailto:algawazy@kku.edu.sa) (Adel M. AL-Ghazzawi) Work Tel: +966-7-241-7041, +966-5-32455786, Fax: +966-7-241-7637. Current address: Department of Chemistry, King Khalid University, Abha, 61413, Saudi Arabia

A.S.a.8

C13CPD CDCl3


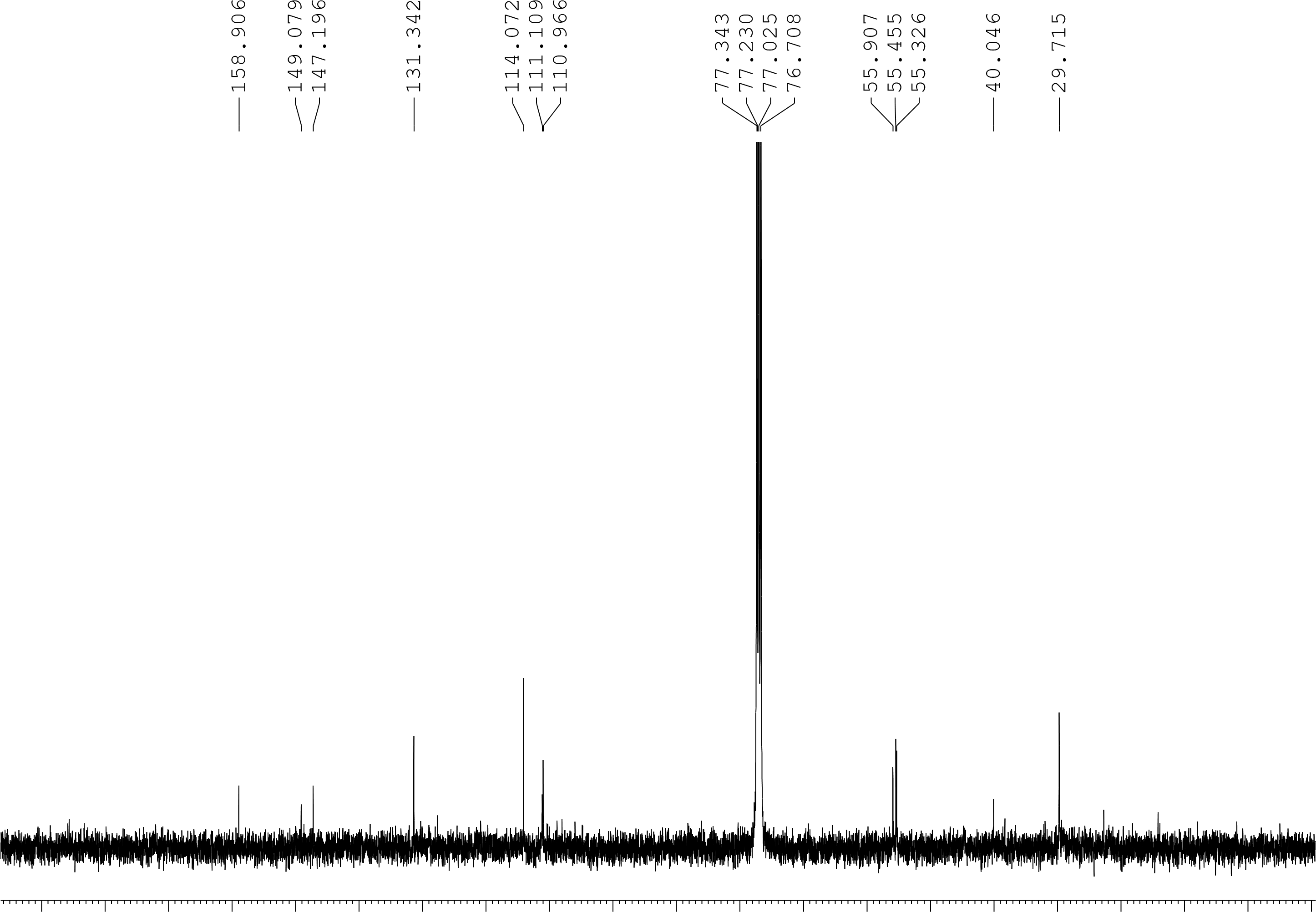
190180170160150140130120110 100 90 80 70 60 50 40 30 20 10 0ppm

Figure S1: ^13^C-NMR spectra of 6, 7-dimethoxy-1-(α-hydroxy-4-methoxybenzyl)-2-methyl-1, 2, 3, 4-tetrahydroisoquinoline

A.S.a.8

^C13DEPT90 CDCl3^


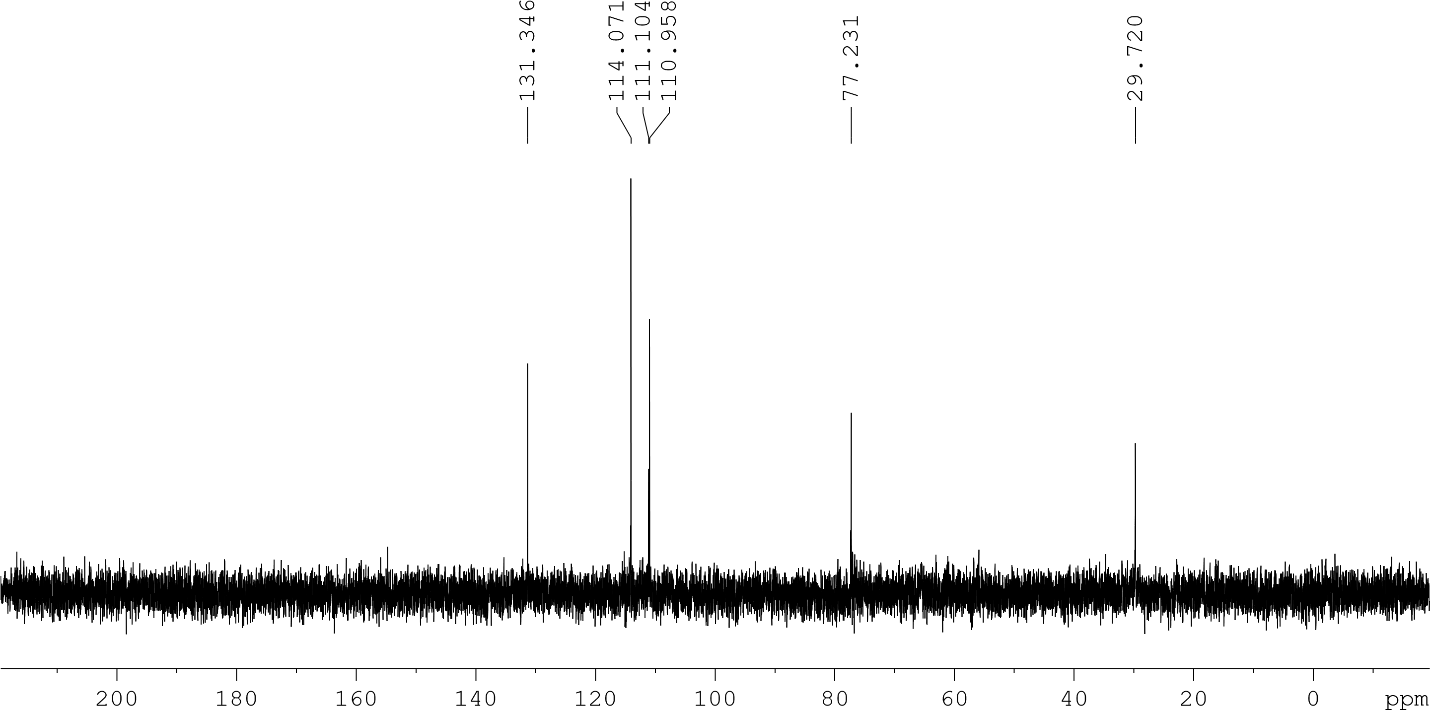
A.S.a.8

Figure S2: ^13^C-DEPT90 spectra of 6, 7-dimethoxy-1-(α-hydroxy-4-methoxybenzyl)-2-methyl-1, 2, 3, 4-tetrahydroisoquinoline

C13DEPT135 CDCl3


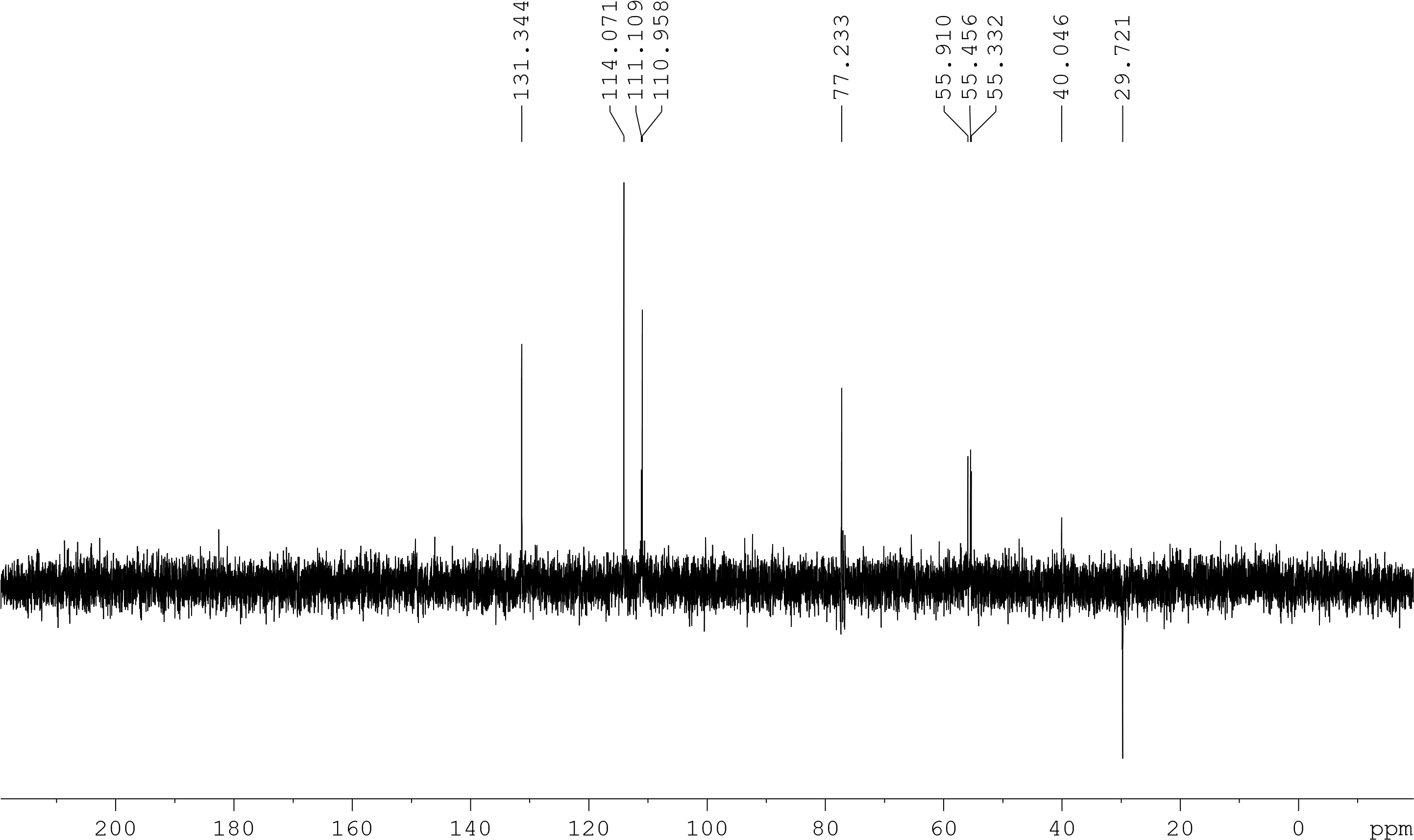


Figure S3: ^13^C-DEPT135spectra of 6, 7-dimethoxy-1-(α-hydroxy-4-methoxybenzyl)-2-methyl-1, 2, 3, 4-tetrahydroisoquinoline

A.S.a.8

PROTON CDCl3


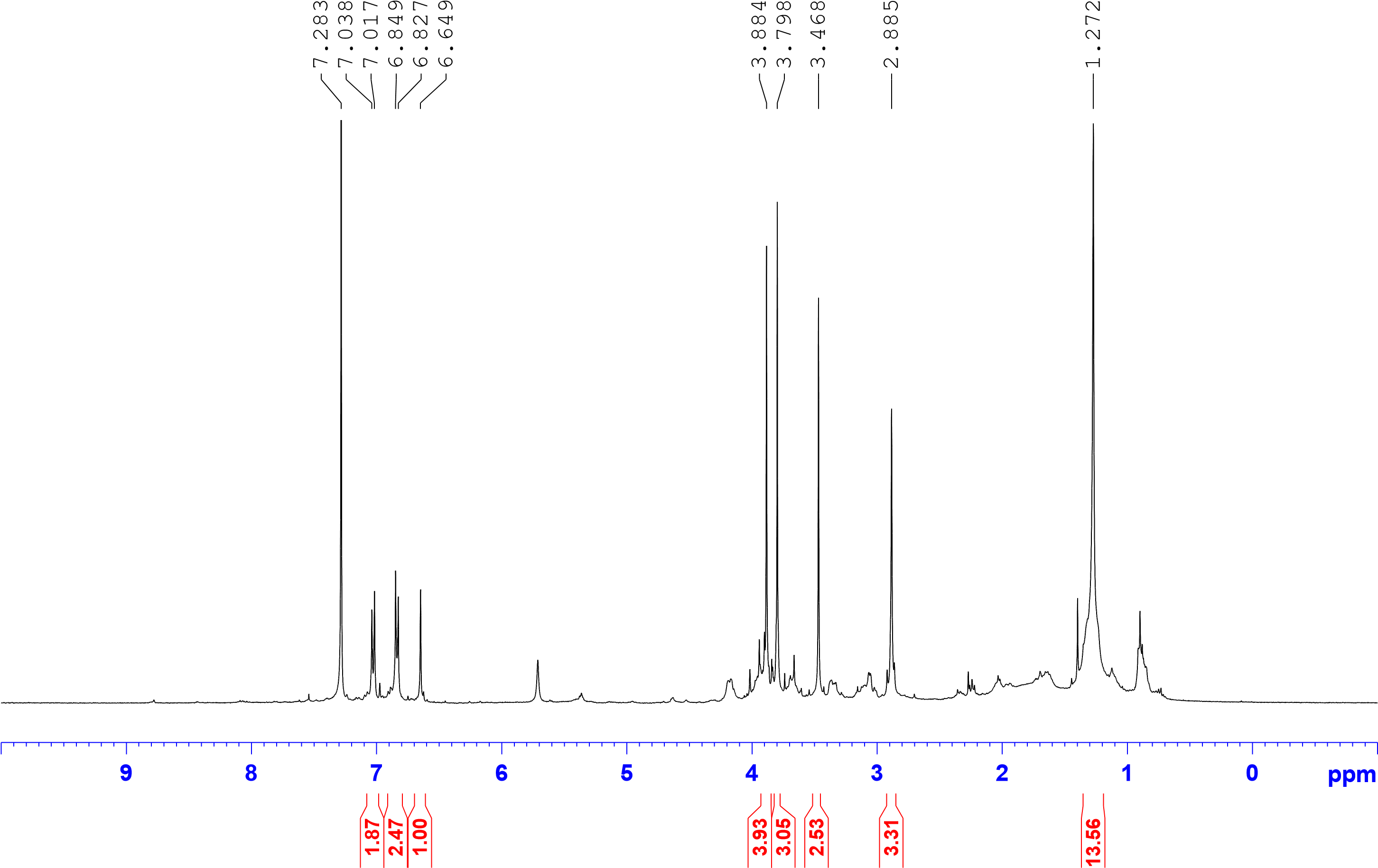


Figure S4: ^1^ H-NMR spectra of 6, 7-dimethoxy-1-(α-hydroxy-4-methoxybenzyl)-2-methyl-1, 2, 3, 4-tetrahydroisoquinoline

A.S.a.14

C13CPD DMSO


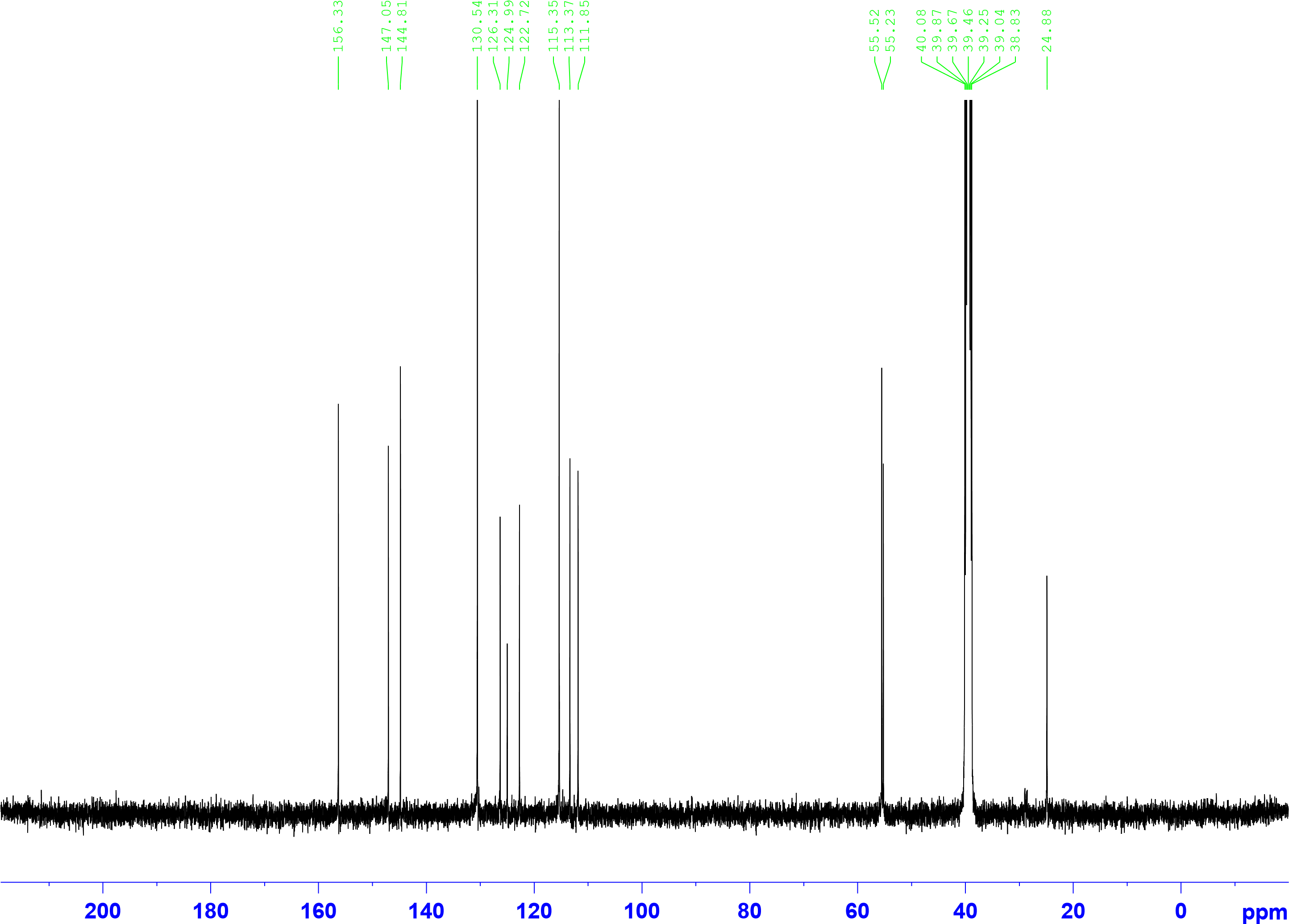


Figure S5: ^13^C-NMR spectra of Coclaurine

A.S.a.14

C13DEPT90 DMSO


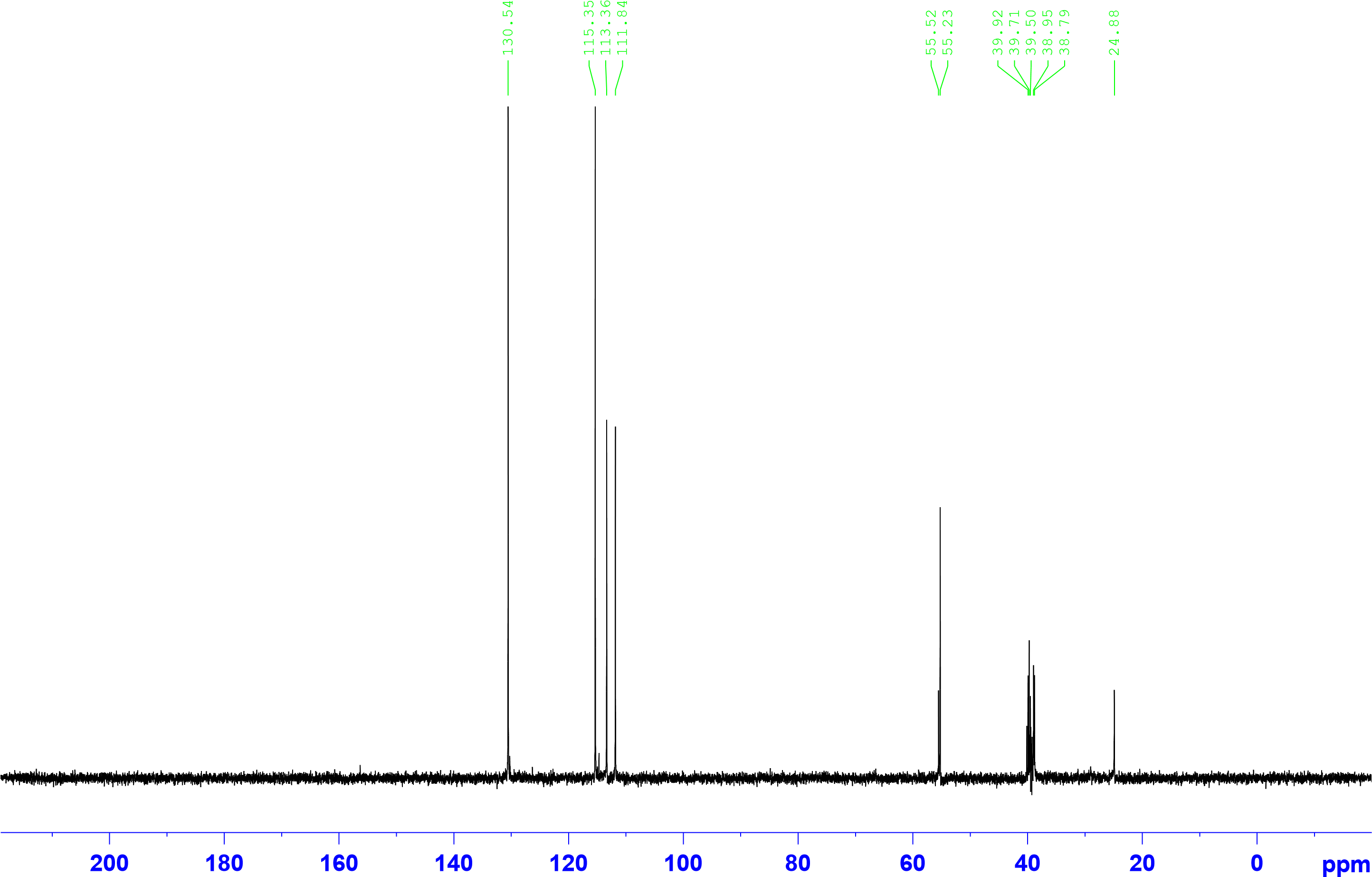


Figure S6: ^13^C-DEPT 90 spectra of Coclaurine

A.S.a.14


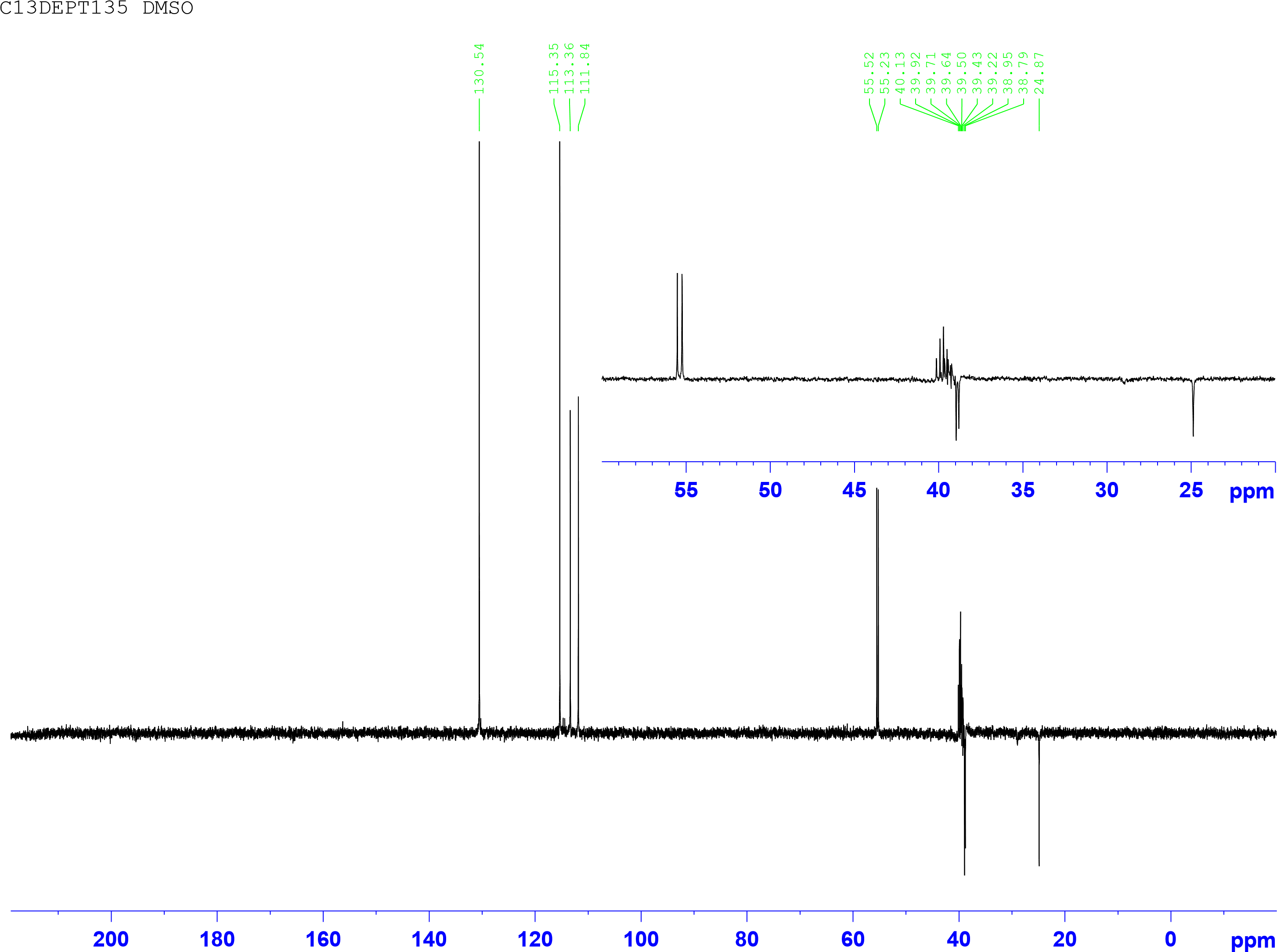


Figure S7: ^13^C-DEPT 135 spectra of Coclaurine

A.S.a.14


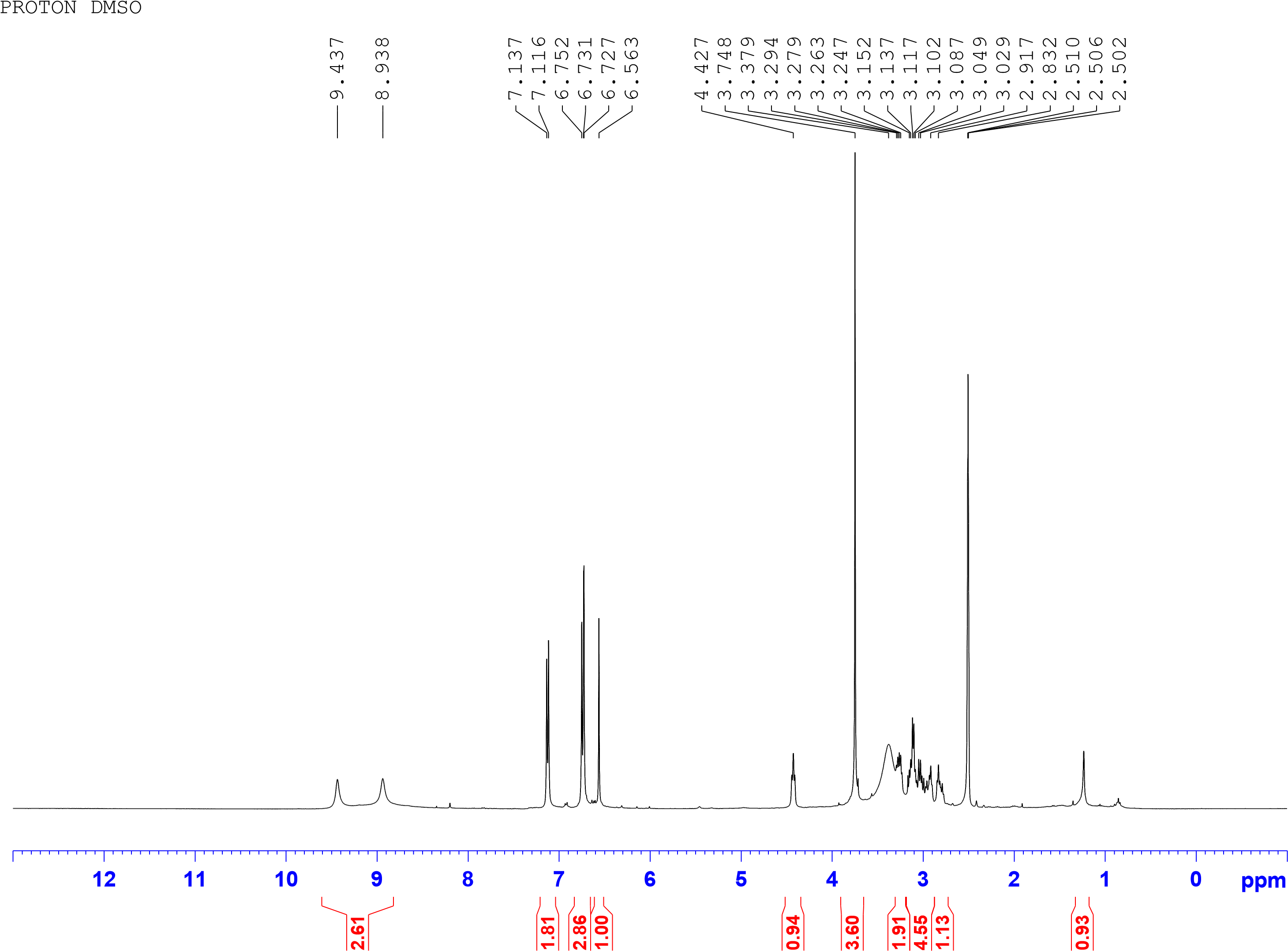


Figure S8: ^1^H-NMR spectra of Coclaurine
